# Observations on Strangulated Scrotal Hernia

**Published:** 1803-03-01

**Authors:** 

**Affiliations:** formerly Surgeon to the Army


*Observations on* STRANGULATED SCROTAL HERNIA, *by Cit.* FARDEAU, *formerly Surgeon to the Army; read at the Medical Society of Paris, 23 Frimaire, (14 Dec.) 11th Year.*

THIS Hernia was so large that it descended half way down the thigh. After vain efforts to reduce it by the *taxis*, Cit. Fardeau had immediate recourse to the operation, which he performed in the following manner. After dividing the integuments and layers of cellular membrane which presented themselves to his bistoury, he perceived a kind of vascular ribbon, which he feared to divide, from an apprehension of hæmorrhage; this band was an inch broad, and came from the posterior and external part of the tumour, and which surrounded it in a spiral direction, running from without inwards until it descended to the bottom of the sac. The operator conjectured rightly, that this might be the spermatic cord, and of which he was assured on pressing with his fingers, when he perceived the resistance of the vas deferens. The pain of which the patient complained, and which was similar to that produced by pressing the cord of the other side, left him no room to doubt the fact. Continuing his operation with the precaution which this circumstance made necessary, he divided *a first sac*, (as he expresses it) at the inferior extremity, and from which issued half a glass of water; this facilitated the return of the tumour as soon as he had dilated the ring by means of a bistoury and director. The second sac was opened to the ring, and from from which issued more water; the contents were then returned into the abdomen, and the testicle and cord resumed their former position. Cit. Fardeau does not give the result of his operations, we cannot therefore say whether the patient has survived. Cit. F. computes that the spermatic cord was at least six times its natural length. He observes, and with truth, that if he had been content with dissecting carefully the cellular membrane at the superior part, in order that he might extend his incision upwards with less precaution, as some recommend, he should have separated the testicle, and performed two operations instead of one. This case offers two facts excessively interesting, namely, the length and disposition of the cord. We know that generally, in the case of scrotal hernia, the spermatic cord is placed posteriorly; instances of a different disposition may be found in Le Dran, (Traitê des Operations), Sabatier (Medicine Operatoire); but we believe this to be the single instance where the cord observed a spiral direction. In the hands of a less experienced surgeon, the spermatic cord might have been taken for some other appearance. We find in the 35th Number of the Medical Journal of Paris, the history of an entero-epiplocele, where it was necessary to cut through several bands which the peritonæum formed, and which pressed on the intestine; three days after the testicle was found mortified; in this case there is a strong probability that the cord was divided. Cit. Fardeau tells us, he freed amply *(largement)* the intestine at the ring; he does not say how he did it, though his expressions would suggest the idea that he deviated from the usual mode. We think it necessary to remark, that the expression, *a first sac*, which the author uses, is not correct, in as far as we should apply the term sac solely to the peritonæum including the protruded part. It is not an unfrequent occurrence to meet with small collections of water in cysts formed by the cellular membrane, and which we presume to have been the appearance which Cit. Fardeau describes; and this is more frequently the case in large hernias of long standing; De Haen (ratio medendi) tells us of a case when more than twenty layers of cellular membrane were divided before the operator came to the proper sac; the interposition of water, so as to form the appearance described, cannot be considered wonderful.

